# Supplementary material for: G4mismatch: Deep neural networks to predict G-quadruplex propensity based on G4-seq data
Source: PLoS Comput Biol. 2023 Mar 10;19(3):e1010948. doi: 10.1371/journal.pcbi.1010948 (PMC10079223; doi:10.1371/journal.pcbi.1010948)
Supplement: S1 Table — (PDF) [file pcbi.1010948.s001.pdf]

Species	K^+	K^++PDS
Homo sapiens	372,627,364	377,015,360
Mus musculus	315,325,949	316,404,581
Danio reiro	165,668,249	152,358,421
Drosophila melanogaster	15,870,402	15,899,380
Caenorhabditiselegans	13,365,121	13,160,158
Saccharomyces cerevisiae	1,589,250	1,551,955
Leishmania major	4,338,042	4,342,208
Plasmodium falciparum	1,828,562	1,657,136
Arabidopsis thaliana	15,332,638	15,335,473
Escherichia Coli	603,254	603,299
Rhodobacter sphaeroides	597,896	600,911
Trypanosoma brucei	2,731,786	2,735,227
