# Supplementary figures and images for: G4mismatch: Deep neural networks to predict G-quadruplex propensity based on G4-seq data
Source: PLoS Comput Biol. 2023 Mar 10;19(3):e1010948. doi: 10.1371/journal.pcbi.1010948 (PMC10079223; doi:10.1371/journal.pcbi.1010948)

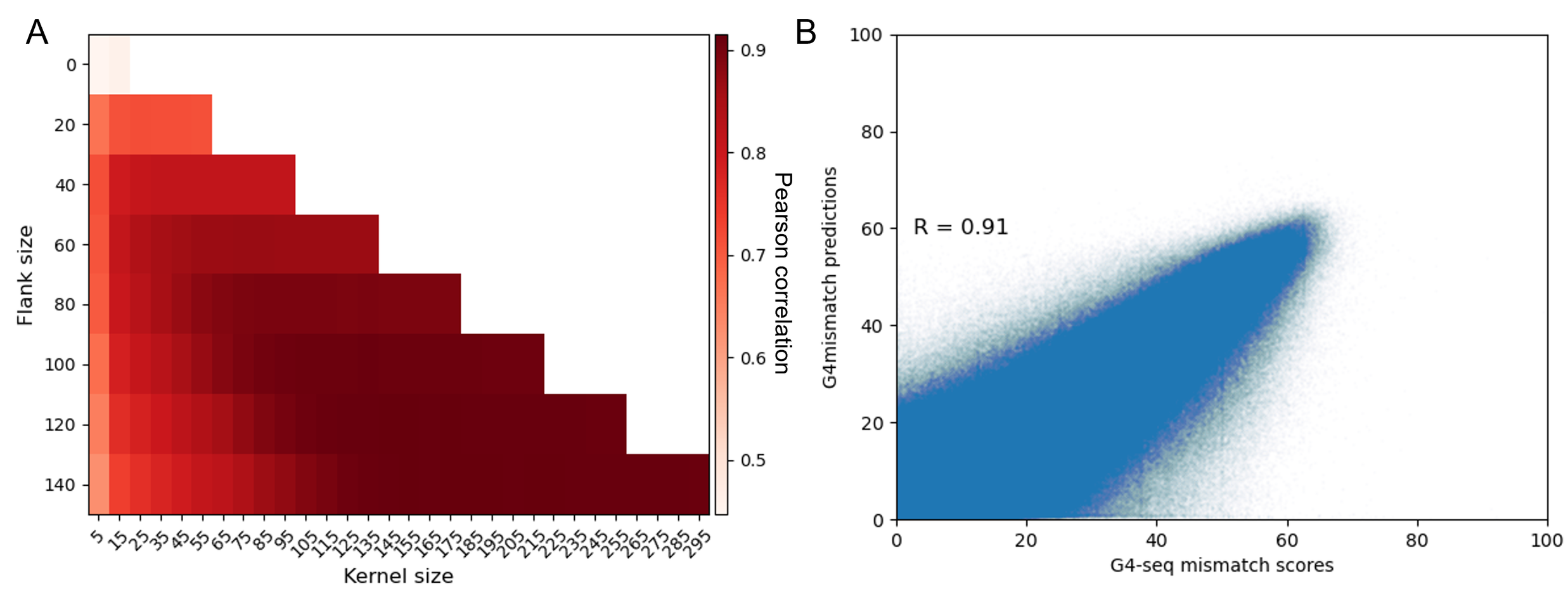

Supplement: S1 Fig — (A) For each flanking sequence length, multiple models were trained differing in their kernel size. Increasing the kernel size resulted in improved performance, which plateaued at a kernel size of the flank length. (B) G4mismatch predictions on chromosome 1 test set highly correlate with G4-seq experimental measurements. (TIF) [file pcbi.1010948.s002.tif]

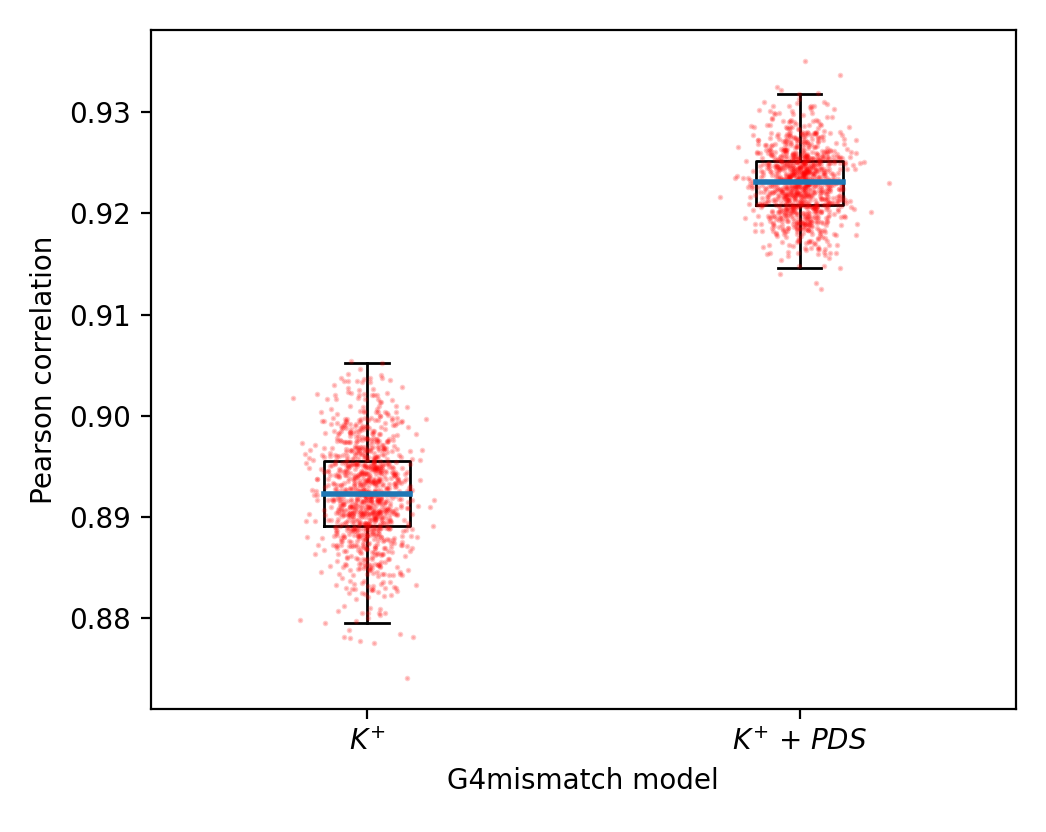

Supplement: S2 Fig — We uniformly binned the held-out test set to 3 bins, and randomly sampled 1,000 samples from each bin over 1,000 iterations to generate balanced test sets. Pearson correlations between predicted and measured mismatch scores over the balanced test sets are reported. (TIF) [file pcbi.1010948.s003.tif]

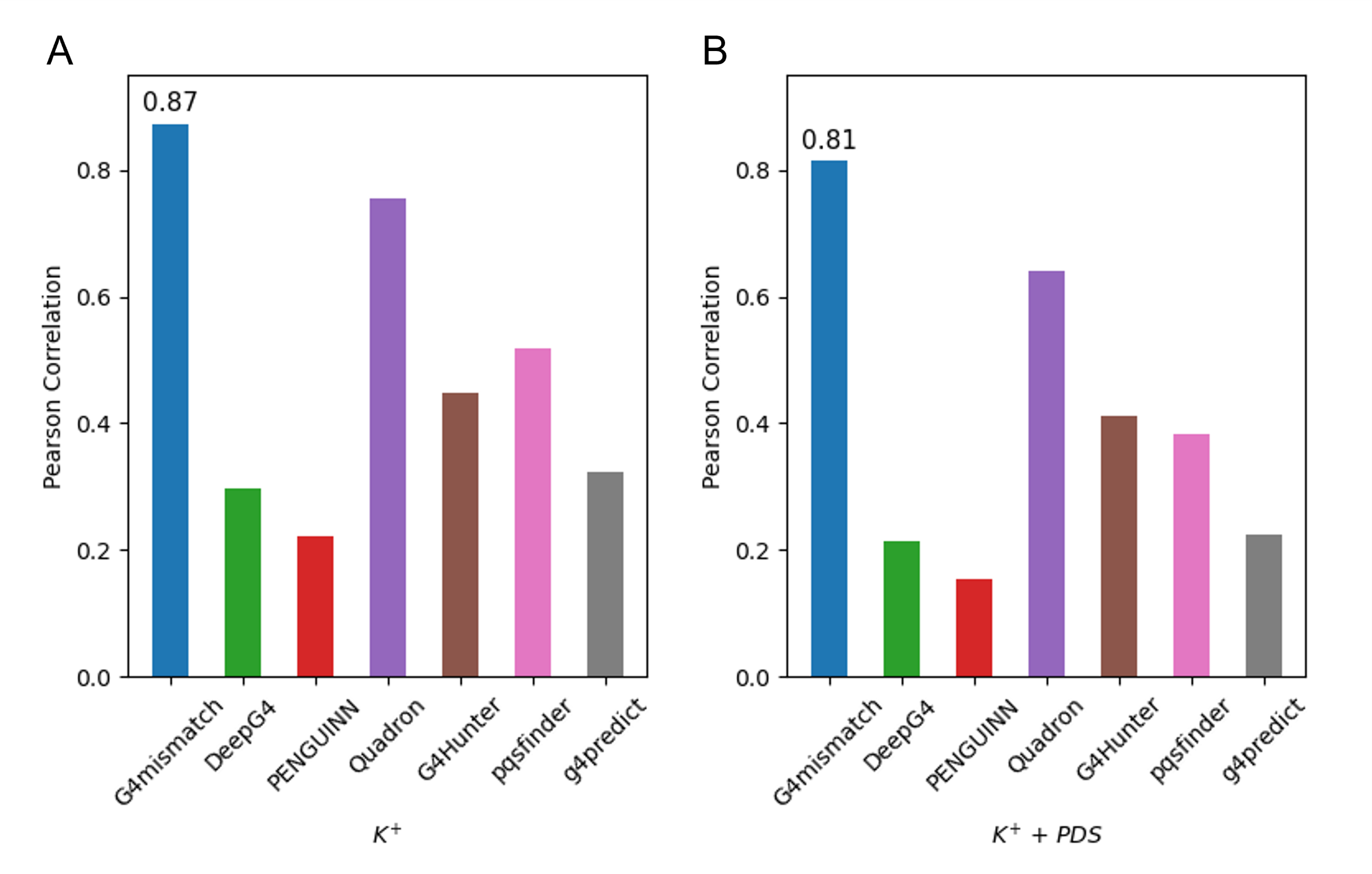

Supplement: S3 Fig — Results on held-out chromosome 1 for (A) K+ and (B) K++PDS stabilizers. We extracted all sequences under the definition of an extended PQ, i.e. {G3+[ACGT]1−12}3+G3+, excluding those which contained PQs within the 100-nt downstream or upstream flanks, and PQs which lack experimental mismatch scores by G4-seq. (TIF) [file pcbi.1010948.s004.tif]

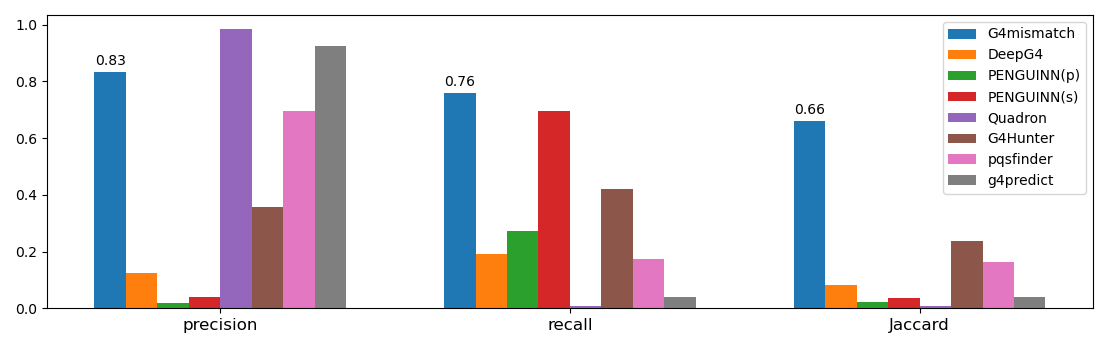

Supplement: S4 Fig — G4mismatch has consistent superior performance as evaluated by precision, recall, and Jaccard scores, achieving high recall without compromising precision. (TIF) [file pcbi.1010948.s005.tif]

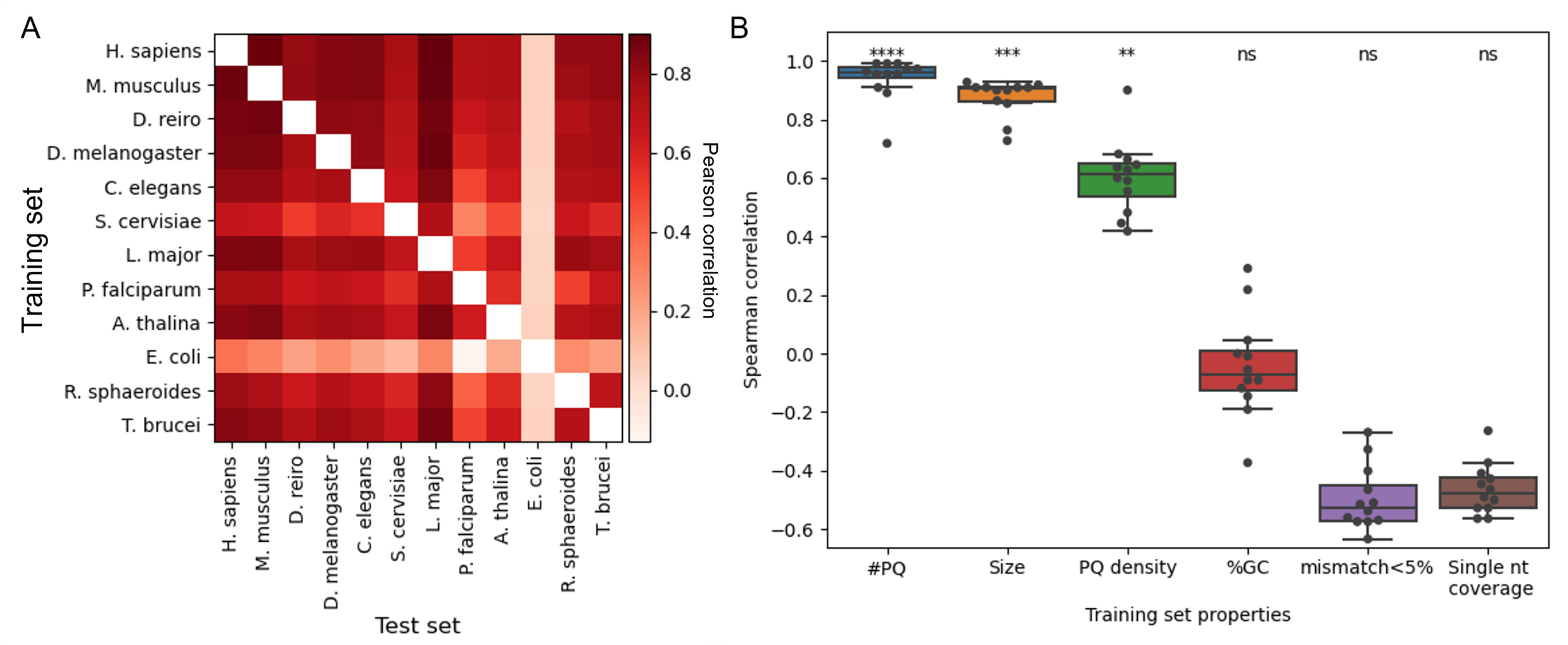

Supplement: S5 Fig — (A) Inter-species G4mismatch K++PDS prediction performance. Each model was trained on data of one species, and tested on all other species. Prediction performance is reported in Pearson correlation of predicted and measured G4 mismatch scores. (B) Correlations between G4mismatch models and number of PQs (#PQs), genome size, PQ density, GC-content (%GC), frequency of mismatch scores under 5%, and single-nucleotide coverage of the datasets each model was trained on. For each property, prediction results were Spearman-correlated to model performance in Pearson correlation. P-value notation: ns = non-significant, ** ≤ 0.009, *** ≤ 0.001, **** ≤ 0.0001, adjusted for harmonic mean p-value. (TIF) [file pcbi.1010948.s006.tif]

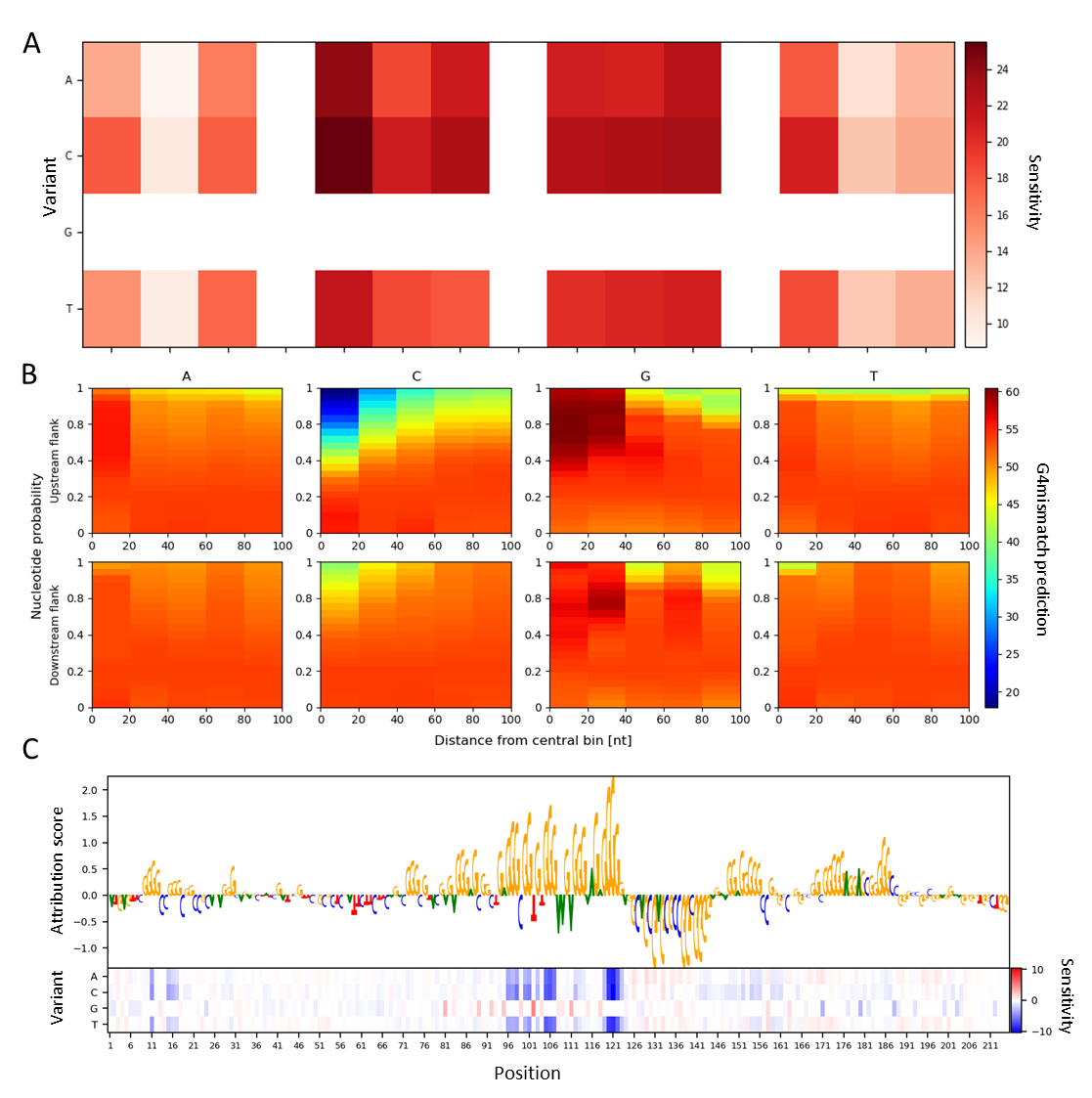

Supplement: S6 Fig — (A) Effect of mismatch in the G-tracts on K++PDS model predictions. For the canonical G4 GGGNGGGNGGGNGGG, we mutated each nucleotide in the G-tracts separately to all other nucleotides, and calculated the change in the predicted mismatch score. (B) Effect of nucleotide composition in the flanking sequences on K++PDS model predictions. For the canonical G4 of the form GGGNGGGNGGGNGGG, we varied the probability of each nucleotide at a time, while assigning uniform probabilities to the other three nucleotides, in 20nt-long regions away from the central bin. We predicted the mismatch score for each such variant, on both the upstream and downstream flanks, separately. (C) The mutation map shows the sensitivity of the K++PDS model to mutations and the corresponding attributions report the importance of a given feature to the model’s prediction. (TIF) [file pcbi.1010948.s007.tif]
