# Supplementary material for: G4mismatch: Deep neural networks to predict G-quadruplex propensity based on G4-seq data
Source: PLoS Comput Biol. 2023 Mar 10;19(3):e1010948. doi: 10.1371/journal.pcbi.1010948 (PMC10079223; doi:10.1371/journal.pcbi.1010948)
Supplement: Attachment — Submitted filename: response_letter.pdf [file pcbi.1010948.s008.pdf]

Reviewer #1: I would like to thank the editor for inviting me to review this research article, titled G4mismatch: Deep Neural Networks to Predict G-Quadruplex Propensity Based on G4-seq Data

The article is well written. The authors have optimised the G4mismatch tool for the best performance by fine-tuning different parameters. It uses existing G4s data for 12 species to train deep neuronal networks and evaluate them against available other data from another research project and cross-species comparisons (trained with one species and tested with another species). The article also reports on the effects of the length of loops, nucleotide composition in flanking regions and mismatches in G4 structures on the predictability of models under two different conditions K+ and K++ PDS. Authors report that G4mismatch performs better compare exiting tools.

Minor comments

1. Figure 7a: In Annotation both K+ and K++PDS are presented by `--` (dashed lines). I guess, dashed line belong to K++PDS.

We thank the reviewer for pointing out this error in our original manuscript. In response to the comment, we corrected the legend of Figure 7A to note that K^+ is annotated by a continuous line while K^++PDS is annotated by a dashed line.

2. It would interesting see the comparison with other new tools like g4predict (Pubmed id: 34650044) and DeepG4 (Pubmed Id: 34383754).

We thank the reviewer for bringing to our attention to these new methods, which we missed in our original manuscript. In response to the comment, we added comparisons to these methods in our manuscript. We mention these methods under the Introduction. We explain how we ran these methods under “Running extant methods for G4 prediction”. We present the results of their prediction performance under “G4mismatch prediction performance evaluation on a held-out test set” and “Comparison of G4 detection performance to extant methods”. We also added the results to Fig 5 and Fig S3 and S4.

3. Please include example datasets in the G4mismatch git-hub repository.

The repository had originally included a shell script called `prep_data.sh` to generate the input data for G4mismatch. In response to the comment, we added an example input file to the GitHub repository.

Reviewer #2: In this manuscript, the Authors propose a CNN-based approach to predict mismatch scores in a genome-wide manner, which would be useful in detecting G-quadruplex structure formation. The Authors used data coming from G4-sequencing experiments, where the mismatch scores were measured for each mapped 15 nucleotide bin throughout the genomes of multiple species.

Major comments

1) In numerous places in the work, the Authors bring up "… no other method was developed for genome-wide prediction of mismatch scores". Note, that a number of (at least three) methods developed before, utilized genome-wide G4-seq data, but surely none that would then predict mismatch scores throughout the whole genome, rather than at only G-rich sites.

We apologize for the confusing phrasing in our original manuscript. In response to the comment, we rephrased the text to note that G4mismatch is the first to be trained on whole-genome mismatch scores, and the first to predict a mismatch score to any 15 nt-long genomic sequence.

The contrast that this work brings by focusing on all the genomic locations to predict mismatch scores, is however dangerous, and should be thoroughly reconsidered or validated by means other than brought in the work.

The human genome contains ~200 times more 15nt bins that do not form G4s, as compared to the bins that take part in G4 formation. The underlying experimental methodology (G4-seq) is based on erratic, unclea, base calling behavior that happens when the polymerase stalls. The resulting errors, that manifest as mismatch scores, are very much dependent on how a sequencer treats uncertainties upon stalling, and how the base callers behave in such conditions. This behavior could be arbitrarily dependent on the exact sequencer model, base caller software version, and conditions, and is not something that one would normally wish to predict. G4-seq experiments, however, then go on to calibrate the methodology from a given experiment, to find the G4 structures specifically, differentiating those signals from the underlying "noise". The proposed G4mismatch model nearly "aims" to predict that underlying noise (with ~200 times more data for that background noise, as compared to actual G4 signals). I am not sure how useful this model in general would be, and how consistent it would be if the sequencer model in G4-seq experiments change.

We thank the reviewer for bringing up this important issue. We agree that as many other sequencing-based experimental techniques, G4-seq results might vary under different experimental settings. However, some of the experimental artifacts and biases are mitigated by normalizing each measurement under a G4-stablizing condition by its measurement under a G4-destabilizing condition. One of our goals is to computationally

simulate the exact conditions that led to the results generated by the G4-seq experiment, which is considered as the state-of-the-art experimental assay to measure G4s. Then, the same methods used to calibrate the methodology from a given experiment in the G4-seq study can be applied to the predictions by G4mismatch. In response to the comment, we discuss this issue in the first paragraph under the Discussion section.

Moreover, the 200-times-more noise part of the genome will mostly have near-0 mismatch scores, as compared to around 40 mismatch scores for actual G4 signals (see y-axis in Figure 4). This type of tight clustering of values at around 0 and 40 will result in artificially high correlation coefficients, hence I would be very cautious in evaluating any of the model performance and validation outcomes from such an unbalanced data representation. While comparing multiple species, the size of the genome might be an important determinant in the success of comparisons (see Figure 6b), by simply increasing the stretch of near 0 mismatch scores.

Indeed, we agree with the reviewer that G4-seq datasets are highly positively skewed mismatch scores. But, this skew is an accurate representation of the endogenous environment. Our aim is to not only accurately predict high mismatch scores, but also low ones to avoid false positives in the G4 detection task. The large number of near-zero mismatch scores is needed to reflect the diversity of non-G4 sequences, which are much more diverse then G4 sequences. Moreover, the genome size in the inter-species analysis refers to the genome size of the training set and on the test set, so it does not affect the difficulty of the prediction task.

In response to the comment, we tested how well does G4mismatch perform under balanced conditions. We uniformly binned the mismatch scores of the test set into 3 bins. We next performed 1,000 iterations, where in each iteration we generated a test set by randomly sampling 1,000 samples from each bin to balance the ratio between high, medium, and low mismatch scores. At each iteration we calculated the Pearson correlation between predicted and experimental G4 mismatch scores and observed a mean correlation of 0.892±0.005 and 0.923±0.003 for K^+ and K^++PDS, respectively (compared to 0.84 and 0.91 achieved on the original test set). We added the results over the balanced test set under “G4mismatch prediction performance evaluation on a held-out test set” and as new Fig S2.

In addition, to show that G4mismatch outperforms existing methods even on a G4-only dataset, we tested G4mismatch’s ability to predict mismatch scores of canonical G4-forming sequences ($\{G^{3+}[ACGT]^{1\text{-}12}\}^{3+}G^{3+}$) and compared prediction performance to existing methods. The results, presented under “G4mismatch prediction performance evaluation on a held-out test set” and as new Fig S3, show that G4mismatch correlates highly with experimental results, achieving a correlation over 0.8, and outperforms existing methods. This result under-scores G4mismatch’s ability to accurately predict high-scoring mismatch scores despite the fact that it is trained on a skewed distribution of mismatch scores.

Moreover, we added the percentage of mismatch scores under 5% as another training genomic feature to test its effect on prediction performance under “Inter-species prediction performance evaluation”. This feature had a relatively low and non-significant average correlation of -0.28 and -0.41 for K^+ and K^++PDS, respectively, which indicates

that the number of low mismatch scores has a negligible effect on prediction performance. We present these results in Fig 6B and Fig S5B.

Last, we added the rational to our choice of the Pearson correlation metric to evaluate prediction performance under “Training G4mismatch models”.

2) As the core validation methodology in the method development and hyperparameter optimization, the Authors used leave-a-chromosome out approach. This however is not correct taking into account a large amount of sequence-wise similar G4s and repeat elements shared between chromosomes. Therefore, there can be a very large amount of sequences in the left out chromosome 2, the core (most influential central 15nt and peri 15-nt) part of which would be very close to the sequences already present in the training set. The training set and the machine learning model, in this setup, could simply overfit the data, arriving to a wrong selection of hyperparameters presented in the work. Later, in the caption of Figure 3 we have “Increasing the kernel size resulted in improved performance, which plateaued at a kernel size of the flank size”. This could mean that no extra abstraction of the sequence was necessary in this setup, and just directly using the original sequence information was resulting into the best performance. Could this be because of the leave-a-chromosome-out validation?

We thank the reviewer for bringing up this issue. Leave-one-chromosome-out is a well-used technique to validate and evaluate prediction performance of machine-learning methods in genomics, including transcription-factor-binding prediction (Won et al. Genome Biology 2010, Zhou et al. IEEE Transactions on Computational Biology and Bioinformatics 2019), pre-miRNA prediction (Raad et al. Bioinformatics 2021), 3’UTR-element prediction (Siegel et al. G3 2022), among many more. In addition, we note that G4s are likely to share sequence similarity as all G4s contain G-tracts with variable loops, so we cannot exclude sequence-similarity effects.

In response to the comment and to alleviate concerns of test-train data leakage, we tested how many 215 nt-long sequences are shared between the test chromosome, chromosome 1, and the chromosomes serving as a training set. Our results show that the number of shared 215 nt-long sequences are negligible: only 0.3% out of all 215 nt-long sequences in chromosome 1. Thus, we are confident that any test-train data leakage effect in the evaluation of the methods is negligible. Regarding the choice of kernel size, since there are only 256 kernels, they are limited in the number of sequences they can ‘memorize’ compared to the millions of 15 nt-long bins appended by flanks generated from each chromosome. We added the rational of our choice to evaluate the methods in a leave-one-chromosome-out setting under “Whole-genome mismatch score prediction evaluation”, and comments on the negligible shared portion of chromosome 1 with all other chromosomes and on the hyper-parameter choice under “Flanking sequence length and kernel size effect on mismatch score prediction performance”.

3) Part of the work discusses the importance of the flanks, and the hierarchy of the 5’ and 3’ flanks in defining the mismatch scores. However, the results have little genomic and G-quadruplex relevance, considering that by flanks the Authors meant flanks near a

15nt stretch, rather than flanks near exact G4 structures. The chosen flank ranges and 5'/3' hierarchies may therefore reflect more of their influence on “background-noise” mismatch scores, and the hierarchy interpretation would be impeded by understanding how much each of those flanks bring the remaining bits of G4 sequence, normally longer than 15nt.

We apologize for the unclear description of the effect of flank size in our original submission. We agree with the reviewer that the effect of the flanks may be partially induced by the G4-seq experiment and the processing of its data by 15 nt-long bins. In the context of our work, the flank size does not directly relate to G4s, but rather to the information needed for the model to make an accurate prediction of the mismatch scores. In response to the comment, we rephrased the text under “Flanking sequence length and kernel size effect on mismatch score prediction performance” describing the effect of the flank size, and other parts of the text referring to the flanks.

Moreover, we note that in the flanking sequence composition analysis (under “G4-folding principles learned by G4mismatch”), we originally designed a canonical G4 sequence to be of length of exactly 15 nt (GGGNGGGNGGGNGGG), and thus any flanking effect in this analysis relates to the flanks of the G4 in its biological relevance.

Further comments

a) The work will benefit from language revision and correction of numerous typos. Examples are “mismatch the scores” (Abstract), should be “mismatch scores”, “quadruplexs” (Introduction), G4hunter, should be “G4Hunter” as elsewhere (Introduction), “gneome” and “memeory” (section 2.3) and so on.

We apologize for these errors and typos. In response to the comment, we went over the manuscript and corrected all typos, including those pointed out by the reviewer.

b) Please, replace “as” in G4s are formed “in the gnome as Hoogsteen bonds between guanines…”, with “facilitated by Hoogsteen bonds” (Abstract and Introduction).

We thank the reviewer for correcting our phrasing. In response to the comment, we replaced the word “as” by the phrasing suggested by the reviewer.

c) In the Author Summary, we can read:
“Previous methods to predict G4s did not take advantage of the genome-wide information provided by the G4-seq experiment, but rather simply solved the problem of G4-folding as binary classification. Our new approach, G4mismatch, is the first to utilize millions of G4 mismatch scores …”
This statement is not true, as a number of machine learning-based methods exist that are based on G4-seq, in part discussed in the same manuscript.

We apologize for the inaccurate description in our original submission. In response to the comment, we modified the sentence to “Previous methods to predict G4s simply solved the problem of G4-folding as binary classification or focused on putative

quadruplexes rather than predicting the raw genome-wide scores generated by the G4-seq experiment.”

Reviewer #3: Thank you for the opportunity to review the manuscript: “G4mismatch: deep neural networks to predict G-quadruplex propensity based on G4-seq data". G-quadruplexes have been demonstrated to be important NA structures required for regulation of basic biological processes. Although there are many algorithms for their prediction, the characterization of active G-quadruplexes remains a challenge. Therefore, new approaches to address this issue are very desirable and welcome. The manuscript contains interesting data; however, crucial citations are mission into the Introduction. The Discussion is very short and unappropriated. The comparisons between species are very interesting; however, the raw data are not presented in the results, so it is impossible to verify authors statements.

We thank the reviewer for the constructive comments. Below, we respond in detail.

Where are the raw data analysis? Where is the overlap of individual sequences with the experimental data? Provide the list of sequences identical to G4-seq, what are different - they are not in G4seq datasets and vice versa?

We apologize for the confusion raised by the unclear description in our original manuscript. The raw data analyzed in our study was generated by the G4-seq experiment. It is publicly available at the NCBI Gene Expression Omnibus (GEO) under accession number GSE110582. No further processing was performed on the data. In response to the comment, we now clearly state this information under “G4-seq data”. We also note that our GitHub repository (https://github.com/OrensteinLab/G4mismatch) had originally included `prep_data.sh`, a script to retrieve the data from NCBI GEO, and to reformat the data to the format expected by G4mismatch. In case the comment refers to the microarray data, there are no shared sequences with the G4-seq dataset. In response to the comment, we now clearly state that under “G4-binding microarray data”.

The G4mismatch score is not described accordingly. There is described only as a comparison to experimental data, but how is used to determine in uncharacterized sequence? What do the numbers on the y-axis in Figure 2b mean? What are the theoretical maximal and minimal scores of G4mismatch?

We apologize for the unclear description of the G4mismatch score in our original manuscript. The mismatch score was originally defined by the developers of the G4-seq assay to represent the ratio in percentages between the number of mismatched base calls observed under a G4-stabilizing condition compared to control conditions over the length of the complete sequence. Thus, its range is 0% to 100%. In response to the comment, we added a description of the calculation and range of the G4 mismatch score under “G4-seq data”, and clearly note that G4mismatch predicts a mismatch score under “G4mismatch neural network architecture”, which explains the meaning of the y-axis in Fig 2B. Sequences uncharacterized by the G4-seq experiment, such as the independent

G4-binding microarray data used in this work for validation, are padded with 100 nt on each side (chosen flank size), and broken into sub-sequences of 215 nt (the input size to G4mismatch). The original sequence is eventually assigned the maximum predicted score, as we originally described under “G4mismatch prediction performance evaluation on an independent microarray dataset”.

I cannot see the mention of 'unique visualization' (?) – Show and describe your 'unique visualization' with examples for various sequences. (chapter 2.7)

We thank the reviewer for pointing out this unclear description in our original manuscript. In response to the comment, we added a discussion of the uniqueness of our visualization approaches in the last paragraph under the Discussion section.

The discussion lacks analysis of results and comparison with other algorithms, what false positives and false negatives for G4mismatch and with comparison with other tested algorithms?

We apologize for neglecting to discuss the results in comparison to other algorithms. In response to the comment, we added a discussion on this comparison in the second paragraph under the Discussion section.

The crucial current literature according to algorithms for G-quadruplex predictions and about G-quadruplex analyses in various species is missing (e.g. A guide to computational methods for G-quadruplex prediction, Nucleic Acids Res., 2020 Jan 10;48(1):1-15., doi: 10.1093/nar/gkz1097., The regulation and functions of DNA and RNA G-quadruplexes, Nat. Rev. Mol. Cell Biol., vol. 21, no. 8, pp. 459–474, Aug. 2020, doi: 10.1038/s41580-020-0236-x., The Presence and Localization of G-Quadruplex Forming Sequences in the Domain of Bacteria, Molecules, vol. 24, no. 9, May 2019, doi: 10.3390/molecules24091711, Landscape of G-quadruplex DNA structural regions in breast cancer, Nat. Genet., vol. 52, no. 9, pp. 878–883, Sep. 2020, doi: 10.1038/s41588-020-0672-8, etc.)

We thank the reviewer for bringing to our attention these important studies, which we did not cite in our original manuscript. In response to the comment, we added a reference to these studies in the manuscript.

Please correct typos, for example: Page 4 – second line: „the reference gneome were stored in memeory“

We apologize for these typos. In response to the comment, we went over the manuscript and fixed the typos, including those pointed out by the reviewer.
