# Supplementary material for: G4mismatch: Deep neural networks to predict G-quadruplex propensity based on G4-seq data
Source: PLoS Comput Biol. 2023 Mar 10;19(3):e1010948. doi: 10.1371/journal.pcbi.1010948 (PMC10079223; doi:10.1371/journal.pcbi.1010948)
Supplement: Attachment — Submitted filename: response_letter.pdf [file pcbi.1010948.s009.pdf]

We are very thankful for the time and effort of the reviewers. Below is our detailed point-to-point response to the two reviewers.

Reviewer #1: Authors have improved the manuscript “G4mismatch: Deep Neural Networks to Predict G-Quadruplex Propensity Based on G4-seq Data ”based on reviewer comments. However, I would like to point out some minor issues.

1. On line 74, “ to generate whole-genome G4 maps for 12 species” can be removed.
2. Figure 2 legend, please change “4mismatch” to “G4mismatch”.
3. Figure S1: Please change PDS to K+ + PDS

We thank the reviewer for pointing out these errors in our revised manuscript. In response to the comment, we corrected these errors. Namely, we removed the part of the sentence on line 74, we corrected our method’s name in Figure 2 legend, and modified PDS to K^++PDS in Table S1.

Reviewer #4: Summary:
The authors propose a novel deep learning algorithm to predict the percent of mismatch in G4seq experiment due to the presence of G4 formation on DNA. The article is well written and well supported by experimental results. Comparison with other algorithms shows the superiority of their approach. However, as far as I understood, the main contribution of their algorithm compared to previously published algorithms G4detector and PENGUINN, is that G4mismatch predicts a quantitative signal (the percent of mismatch), whereas the other algorithms predict mismatch peaks (binary classification).

We thank the reviewer for highlighting the conceptual innovation of our method, G4mismatch, which is the prediction of a continuous signal rather than a binary classification as was proposed by previous methods. This innovation was motivated and led to several major advantages of G4mismatch compared to previous methods. By training a regressor to predict a mismatch score we removed the requirement to define an artificial and synthetic negative dataset, which we previously showed has a major effect on the performance and applicability of the trained model. Moreover, by training a regressor we in fact developed a simulator of a G4-seq experiment for any set of sequence of interests, which has many benefits some of which we demonstrated in the manuscript, such as improved G4 detection and systematic and through comparison between species. We conclude that our conceptual innovation led to a breakthrough in predicting DNA G4 propensity scores, which we expect to be useful in many future G4 studies.

Major revisions:
- the authors wrote: "The G4 mismatch score, as defined by the developers of the G4-seq assay, is calculated as the ratio of the number of mismatched base calls observed under a G4-stabilizing condition compared to control conditions over the length of the complete sequence (either mismatched or not). Hence, its range is 0% to 100%."

Later, they wrote: "The network’s output goes through a single neuron with linear activation.", meaning that the model is aimed to predict a value ranging from minus infinity to plus infinity, while the mismatch score actually range from 0% to 100%.
If I understood correctly, I believe a sigmoid activation as output would better suit, since the output is between 0 and 1. If no, the authors must explain this point.

We thank the reviewer for bringing up this important point which we neglected to discuss in our revised manuscript. Indeed, a sigmoid function bounds the output to a range of [0, 1], and is very popular in binary classification problems. In our work, we aim to predict the output of a G4-seq experiment, the %mismatch of a 15nt-long bin, a number which is corelative to the stability of the G4. Since the %mismatch itself is only correlated to G4 stability (and not an absolute value), we are interested in predicting a number which is highly correlated with the %mismatch rather than the exact number. For this aim, we evaluated prediction performance by the Pearson correlation coefficient, which measures the linear agreement between two vectors, indifferent of any linear scaling. This is further demonstrated in the G4 detection task, where only the ranking of nucleotides and an accompanying threshold are important rather than exact values. Similarly, the comparison between species may produce predictions on different scales that depend on the specific experimental details. Hence, evaluating prediction performance by Pearson correlation is appropriate.

We neglected to report in the revised manuscript that a network with sigmoid activation function at the output node performed much worse compared to a network with linear activation at the output node. A model with an output sigmoid activation achieved on the validation set a Pearson correlation of 0.69 and 0.81 compared to 0.82 and 0.91 by the model with an output linear activation for K^+ and K^++PDS, respectively. In response to the comment, we added the rationale and these results under the Methods section.

- In section "Inter-species prediction performance evaluation", the authors wrote that "Results show that some species-specific models were much more accurate than other models". Could it be partly due to the heterogenous quality of data between the different species?

We agree with the reviewer that it is very likely that part of the prediction performance variability is explained by the quality of the experimental data. Among the parameters we were able to extract, we only found one that correlates with experimental data quality, which is single-nucleotide coverage. In response to the comment, on lines 401-402, we clarified the description of the single-nucleotide coverage parameter used to assess the effect of sequencing depth and quality of the dataset on prediction performance, and added that this is the only parameter we could extract that is correlated to experimental data quality. In addition, we revised our hypotheses re reasons for prediction performance variability accordingly.

- the authors explored the impact of mutations on G4 based on their predictions. A simple way for the authors to experimentally validate their findings is to use human GTEx expression SNPs (eSNPs) and check what is the impact of their mutation on expression, given that a G4 in a gene promoter is known to regulate the gene

expression. With my students, we have done already similar analyses, and there is some chance it will reveal some interesting results.

We thank the reviewer for this great suggestion. We believe that since our manuscript already contains method’s development, evaluation, testing, downstream application, and cross-specic analysis, it is best suited for future work. In response to the comment, we added this potential line of research as future work under the Discussion section.

- the authors propose an algorithm that is very similar to previously published algorithms G4detectors, PENGUINN and DeepG4, with a very classical CNN architecture.
Given the recent progress in deep learning, the authors must try to use attention (Enformer) or deep residual CNN (Basenji) to improve their results. For instance, based on my experience, it is very likely that deep residual CNN with for instance 5 layers of CNNs with residual connections combined with dilated convolution with kernel size of 3 (or 5) will significantly improve the results (see Basenji).

We agree with the reviewer that the deep learning field consistently sees exciting development, which have the potential to provide for further improvements in genomic prediction tasks. However, as fine-tuning, testing, and evaluating different neural network architectures is a process that can take a long time, and as our model already achieved a Person correlation > 0.9, we believe that this line of research is out of scope of the current study. In response to the comment, we added this potential line of research as future work under the Discussion section.

Minor revisions:
- "The detected sequences are scored based the length of the tetrads" -> "The detected sequences are scored based ON the length of the tetrads"

We apologize for this error. In response to the comment, we rewrote the sentence as suggested.

- in the intro, you should explain that DeepG4 differs from other CNNs such as G4detector and PENGUINN, since it was trained (and aimed) to predict active G4s, meaning G4s formed in vitro (G4seq) which are active in vivo (G4 ChIP-seq) and thus cell type dependent. It thus explains why DeepG4 has lower accuracy when it comes to predict G4seq results, compared to G4detector and PENGUINN. It was actually explained in the results, line 369-370.

We thank the reviewer for pointing out the need to emphasize the difference of DeepG4 compared to G4 detector and PENGUIN in the Introduction. In response to the comment, we added “but unlike the first two models, which were solely trained on in vitro data, DeepG4 was trained to identify G4s which are active in vivo” in lines 56-57 under the Introduction section.

- in the intro, you should clearly state that G4mismatch predicts G4seq signal as a quantitative regression problem unlike G4detector and PENGUINN which predicts G4seq peak as a binary classification problem.

We apologize for failing to clearly explain the output of G4mismatch. In response to the comment, we added in lines 62-63 that G4mismatch predicts “a quantitative value that correlated with G4 stability” under the Introduction section.

- The authors wrote : "The input to G4mismatch is a one-hot encoded 215 nt-long DNA sequence matrix. N positions in the DNA sequence were replaced by a vector of 0.25 indicating a uniform probability to all nucleotides."
THen they wrote: "The uniqueness in our visualization approaches stems from our choice to
encode our inputs as local nucleotide distributions rather then one hot-encoding of specific sequences."
I don't understand "local nucleotide distributions". Do the authors mean the weight attributed to each nucleotide when assessing its contribution to the G4 formation? Please explain.

We apologize for the confusion caused by our unclear phrasing. In response to the comment, we added this clarification “In this encoding, we varied the weight of the four nucleotides in each position, such that the sum of the weights is one, allowing it to represent a distribution of sequences,” in lines 521-523.
